# The Right to Explanation in AI: In a Lonely Place

**DOI:** 10.2196/64482

**Published:** 2025-09-12

**Authors:** Alycia Noë, Sarah Bouhouita-Guermech, Ma'n H Zawati

**Affiliations:** 1 Centre of Genomics and Policy McGill University Montreal, QC Canada

**Keywords:** automated decisions, automation, clinical decision-making, machine learning, artificial intelligence, algorithm, predictive model, predictive analytics, practical model, deep learning, early warning, early detection, right to explanation, doctor, physician’s obligations, oath

## Abstract

Technology is increasingly being used in decision-making in all fields, particularly in health care. Automated decision-making promises to change medical practice and potentially improve and streamline the provision of health care. Although the integration of artificial intelligence (AI) into medicine is encouraging, it is also accompanied by fears concerning transparency and accountability. This is where the right to explanation has come in. Legislators and policymakers have relied on the right to explanation, a new right guaranteed to those who are affected by automated decision-making, to ease fears surrounding AI. This is particularly apparent in the province of Quebec in Canada, where legislators recently passed Law 5, an act respecting health and social services information and amending various legislative provisions. This paper explores the practical implications of Law 5, and by extension of the right to explanation internationally, in the health care field. We highlight that the right to explanation is anticipated to alter physicians’ obligation to patients, namely the duty to inform. We also discuss how the drafting of the legislation on the right to explanation is vague and hard to enforce. This dilutes the potential of the right to explanation to provide meaningful protections for those affected by automated decisions. After all, AI is a complex and innovative technology and, as such, requires complex and innovative policies. The right to explanation is not necessarily the answer.

## Introduction

With the advancement of technology, we are increasingly turning to computers and machines to streamline our decision-making. This is evident in health care, where automated decision-making (ADM) promises to considerably transform the field. This is already seen in radiology and cardiovascular imaging, where artificial intelligence (AI) computer systems serve as a decision-making support tool for clinicians to streamline their practice; for example, AI imaging tools can perform on par with clinicians in areas such as mammographic screening, potentially allowing more time for patient interactions and research [1,2]. However, with the use of patient data and computers also come fears and worries about patient privacy, the protection of personal information, and transparency in the use of technology. Legislators and policymakers have sought to ensure that the use of ADM is sensitive to the societal values of fairness, justice, accountability, and transparency by legislating the right to explanation. The province of Quebec in Canada recently responded to fears surrounding AI by becoming the first jurisdiction in the country to encode the right to explanation for any automated decision in the health care context, captured by Law 5 [3], an act respecting health and social services information and amending various legislative provisions (see amendments to s 12.1 of An Act respecting the protection of personal information in the private sector in s 110 of Law 25 and s 65 of Law 5 [4]). This paper focuses on the impact of legislating the right to explanation on the provision of health care, using Law 5 as a case study to better evaluate its implications.

From the standpoint of individual protections and rights, the right to explanation is a first step toward legislating true AI explainability and transparency. The right to explanation requires an explanation of automated decisions being given to individuals affected by these decisions. This right is meant to guarantee further transparency in how automated decisions are rendered, allowing for a mechanism of accountability and responsibility. While requiring an explanation sounds like a solution to increase the transparency of ADM, AI explainability faces the challenge of keeping up with complicated, and perhaps inherently unexplainable, technology. There are several types of explanations related to ADM; for example, some explanations are more technical and describe the mechanisms that an algorithm uses to generate an output. In a clinical context, explanations are often transmitted between clinicians and patients to ensure that patients have the information they need to understand their health and make informed decisions about it. AI explainability is a dedicated field focused on making AI systems more understandable, especially given the opaque nature of complex models. This field includes both technical and social aspects, as researchers work to develop mathematical methods for explaining AI decisions while also exploring which explanations are necessary for users and determining their impact to support responsible use [5]. This means that different types of explanations could exist depending on the stakeholders involved, namely developers, health institutions, physicians, and patients; for instance, developers would need to understand how an algorithm works, whereas patients’ focus is directed toward explanations that impact their own health [6]. In this paper, we focus on the right to explanation in the legal context, examining existing regulation on ADM and how it supports this right, affecting multiple stakeholders, using AI in health care as an example. As such, any policy that is implemented must be top notch, meaning that it must be both informed by the technology itself and readily adaptable to the ever-changing nature of ADM.

For this reason, the right to explanation, as it stands today, represents, at worst, an onerous addition to a physician’s duties or, at best, an ineffective protection offered by legislators to quell citizens’ fears around automation. To explore the impacts of the right to explanation on the use of ADM in health care, this paper focuses on Law 5 in Quebec as a case study. This research is presented in three sections. In the Legislating the Right to Explanation section, to set the scene, we discuss the history and the legislation of the right to explanation in Quebec. In the What Constitutes a Good Explanation? section, we explore the essential elements of a good explanation. This is important to understand how mandating an explanation of ADM in health care impacts physicians’ duties and the adoption of these technologies. Finally, in The Effect of the Right to Explanation on Health Care Decision-Making section, we discuss the implications of the right to explanation for physicians’ preexisting duty to inform in Quebec and how relevant the codified right to explanation is to how ADM is currently used in health care services. Through these sections, we assert that while the right to explanation, on its surface, promotes transparency in ADM, it is not an ideal approach, given the complexity and inherent unexplainability of these technologies. As a result, the right to explanation, depending on its framing, either becomes a performative action by legislators to alleviate fears concerning automation or an overly burdensome obligation imposed on physicians that will restrict health care from advancing. There is no middle ground.

While there are hesitations around ADM, it is undoubtedly the future. If used correctly, ADM can improve the quality of health care and contribute to more effective care delivery in Canada. Ultimately, this paper provides a starting point to understand how the right to explanation will be applied to health services that integrate ADM and how this right serves to affect the adoption of new technologies in Quebec and internationally. Furthermore, we address the question of what should be done with the now-legislated right to explanation, given its ineffectiveness.

## Legislating the Right to Explanation

### Overview

As AI and ADM start to be more integrated into our daily lives, the fact that AI cannot be explained is seen as a liability and an obstacle to accountability. Lawmakers and policymakers have begun to realize that they must accordingly update legislative frameworks to respond to these ever-changing realities. The growing use of AI in health care is especially concerning for global lawmakers. The common fears associated with the use of ADM in medicine are patient privacy, the protection of personal information, and the lack of transparency and understanding of ADM systems. The right to explanation attempts to address these concerns.

### AI Used for Medical Imaging Analysis

Before getting into the origins of the right to explanation and what constitutes a good explanation, it is important to understand in what health care settings automated systems are used today. These include, but are not limited to, diagnosis, treatment, and drug development; for example, ADM is increasingly present in the field of medical imaging. Medical images are often central for physicians in making diagnoses and treatment decisions [7]. However, image analysis necessitates a fair amount of a physician’s time, time that is invaluable in modern-day health care systems that are struggling for resources [8]. As such, the analysis of medical images is increasingly being delegated to automated systems. The use of computer-aided diagnosis (CAD) systems in hospitals helps pathologists evaluate complicated medical images more quickly than would be possible manually [9,10]. This idea of integrating computers into medicine is not new; it has just become more advanced with recent technological developments [11]. In fact, CAD was first used back in 1967 by scientists for the detection of abnormalities in mammograms [12]. These systems serve as a built-in second opinion. Rather than a radiologist needing to wait for another colleague’s opinion, the opinion of the computer system serves as the second set of eyes. CAD also ameliorates the analytical routine of radiologists by identifying structures of interest that perhaps they had not noticed themselves [13]. The use of this technology, at its best, can improve mortality rates, increase treatment success, and enable the early detection of diseases [14,15]. While there are several possible benefits of ADM integration into medicine, there is also hesitancy because ADM models often lack transparency, and therefore the classical accountability mechanisms (eg, medical liability and the physician’s standard of care as well as standards of professional competence) in medicine are less effective because they focus on the physicians themselves [16,17], rather than the technological aids they use.

### Origins of the Right to Explanation

Technology is ever changing. This is especially true with ADM models. These models are complicated and hard to understand. While the initial integration of ADM into health care involved relatively simple systems whose decisions could be easily interpreted or confirmed by physicians (eg, interpreting x-rays), the latest ADM technologies are designed to mimic neural networks in the human brain. Scientists are still trying to fully understand how human neural networks function. Therefore, it would be impossible for anyone to fully understand these automated systems that replicate neural networks.

With the prospect of integrating further automation and more complicated ADM systems into society, many legislators sat uneasy with the lack of both accountability and understanding associated with these systems. This is where the right to explanation enters the picture.

The right to explanation is an offshoot of the right to privacy, which was first recognized in 1950 by the European Convention on Human Rights [18] and ensures “respect for [an individual’s] private and family life” [19]. Consequently, each signatory to the European Convention on Human Rights adopted privacy legislation to comply with it. It is in these legislations that we find the origins of the right to explanation for decisions made with automated systems.

The first recognition of the legal right to explanation in relation to ADM occurred in France in 1978. In its initial data protection legislation, the French government included the right to know and challenge the logic used by automated processes [20].

Following France’s example, the European Union (EU) passed the European Data Protection Directive in 1995 [21] to update privacy legislation in a manner that showed consideration of technological advancements. This directive established a minimum level of data privacy and security standards [18]. However, these standards did not anticipate that the internet would morph into what it has become today. The European Data Protection Supervisor acknowledged gaps in the existing privacy protection framework in 2011, declaring an urgent need for “a comprehensive approach on personal data protection.” This statement prompted work on an overhaul of the 1995 directive [22]. This work was slow, but it ultimately led to the EU’s General Data Protection Regulation (GDPR), which came into force in 2016 and has been applicable to all member states since May 2018 [18]. The GDPR recognizes many individual privacy rights under the umbrella of the right to privacy, one of which is the right to explanation. The GDPR is now considered the global template for the right to explanation and undoubtedly served as inspiration for Quebec’s laws. The right to explanation in the GDPR is explained as follows [23]:

The data subject should have the right not to be subject to a decision, which may include a measure, evaluating personal aspects relating to him or her which is based solely on automated processing and which produces legal effects concerning him or her or similarly significantly affects him or her...Such processing includes “profiling” that consists of any form of automated processing of personal data evaluating the personal aspects **relating to a natural person, in particular to analyse or predict aspects concerning the data subject’s** performance at work, economic situation, **health,** personal preferences or interests, reliability or behaviour, location or movements, where it produces legal effects concerning him or her or similarly significantly affects him or her...In any case, such processing should be subject to suitable safeguards, which should include specific information to the data subject and the right to obtain human intervention, to express his or her point of view, **to obtain an explanation of the decision reached after such assessment** and to challenge the decision.

A knowledge of the definitions of the right to explanation worldwide is important to understand the influences on Quebec legislation and possible interpretations of this right in Quebec.

### Law 5 in Quebec

#### Overview

Quebec is the first province in Canada to meaningfully update its privacy legislation to address questions brought forth by recent technological advancements in AI. The use of AI, especially in health care, raises significant privacy concerns because it relies on the collection of large amounts of personal information. As it has now become virtually impossible to interact with an increasingly digitalized and AI-integrated world without sharing personal information, the Quebec legislature recognized the need to update its privacy legislation to reflect present-day realities (see the Journal des débats [Hansard] of the Committee of Institutions, 42-1, Vol 45, No 113 [2 March 2021] at 16h50 [M Caire] [24]). To address this issue in the provision of health care, legislators passed Law 5, an act respecting health and social services information and amending various legislative provisions. Law 5 mandates how every health and social services body must handle the health information it holds [25]. Together, these amendments to Quebec privacy legislation adopt the right to explanation in the province, making it one of the most demanding jurisdictions worldwide regarding data protection [26].

This legislative improvement to Quebec’s privacy regime is a proactive step toward preparing the province for the continued integration of AI into health care and other sectors (see the Journal des débats [Hansard] of the Committee of Institutions, 42-1, Vol 45, No 126 [17 March 2021] at 17h [M Nadeau-Dubois] [24]). No particular AI technology is discussed in this law, as it is intended to be technologically neutral and adaptable to future developments (see the Journal des débats [Hansard] of the Committee of Institutions, 42-1, Vol 45, No 126 [17 March 2021] at 14h40 [M Caire] [24]). By introducing new rights for Quebecers, such as the right to explanation and the right to contest fully automated decisions, Law 5 purports to be an important step away from digital paternalism (see, for example, the discussion of potentially discriminatory bias of AI algorithms in Journal des débats [Hansard] of the Committee of Institutions, 42-1, Vol 45, No 126 [17 March 2021] at 17h50 [M Nadeau-Dubois] [24]). These new rights granted to patients need to be evaluated for their possible effects on the use of ADM in the provision of health care in Quebec, but before doing so, it is crucial to understand some of the outlined goals of the statute, namely transparency and understanding how machines work.

#### Transparency

While the term itself cannot be found in the law, transparency is both a primary and overarching goal of Law 5. This reflects the integration of the right to explanation into other pieces of international legislation. The right to explanation is considered a necessary element for adequate transparency of automated decisions [27]. Adequate transparency requires that any information resulting from automated decisions is clear and easily understandable. Furthermore, it requires that any information used to render this automated decision is easily accessible [23].

The intent, with the Quebec law, is to offer confidence to lay individuals as new technologies (eg, ADM processes) become part of daily life (see the Journal des débats [Hansard] of the Committee of Institutions, 42-1, Vol 45, No 126 [17 March 2021] at 17h50 [M Nadeau-Dubois] [24] “Non, l'idée, c'est de dire : Donc, on met des balises puis on maximise la transparence pour que les gens aient confiance dans ces nouveaux processus là qui vont se généraliser”). Law 5, building on Bill 3, a previous piece of legislation that legislated the right to explanation for automated decisions made by public bodies and private entities, aims to increase the transparency of health care automated processes that involve personal information. An individual can now restrict how their personal information is used, and it is within their purview to refuse the sharing of their personal information with spouses, direct descendants, and researchers [25]. The law also includes provisions for oversight to increase transparency, including inspections by the Commission d’accès à l’information (Access to Information Commission) to verify a body’s compliance with the Law 5 [25] and mandatory publication of governance policies [25].

#### Understanding How Machines Work

Interestingly, the right to explanation has also been asserted as a means to better understand how machines work. (“Machine” is the word used throughout the debates on the floor of the National Assembly to refer to automated technologies. See, for example, the Journal des débats [Hansard] of the Committee of Institutions, 42-1, Vol 45, No 126 [17 March 2021] at 16h50 and 17h20 [M Caire] [24].) This was confirmed in debates concerning the right to explanation, where it was underscored what the right guarantees—an explanation of the nature of the decision, how it was made, and the reserved right to rectify certain elements of the decision (see the Journal des débats [Hansard] of the Committee of Institutions, 42-1, Vol 45, No 126 [17 March 2021] at 17h50 [M Nadeau-Dubois] [24]). It is important to note that the position of the legislators on what constitutes the right to explanation is only persuasive and not legally binding. The definition of the right to explanation has yet to be tested in court. If courts adopt the definition elucidated by the Quebec National Assembly, the idea is that these explanations of automated decisions will lead to a more comprehensive understanding of how ADM works. This definition would also provide more clarity on what constitutes a good explanation and would represent a significant step toward a measurable and enforceable right to explanation. However nice it may sound to have a clear definition of an explanation, the aforementioned definition will likely hinder the development of automated systems. As ADM becomes more integrated into society, it expands and becomes more unexplainable, as much of ADM is performed with algorithms based on the functionality of the human brain, which we still do not fully understand. This is the trade-off for speed and performance [28]. This is why explanations are more often required for high-risk technologies, such as those used in health care, rather than low-risk technologies, such as an AI technology used to identify bird species (see, for example, EU Artificial Intelligence Act, Art 86(1): “Any affected person subject to a decision which is taken by the deployer on the basis of the output from a high-risk AI system listed in Annex III, with the exceptions of systems listed under point 2 thereof, and which produces legal effects or similarly significantly affects that person in a way that they consider to have an adverse impact on their health, safety or fundamental rights shall have the right to obtain from the deployer clear and meaningful explanations of the role of the AI system in the decision-making procedure and the main elements of the decision taken” [29]).

## What Constitutes a Good Explanation?

Given that the right to explanation has been codified in numerous countries, the question arises as to the elements of a good explanation. It is important to understand what the elements of a good explanation are before evaluating the impact of codified rights to explanation on ADM in health care. In the clinical context, the lack of transparency in automated decisions has been a significant challenge to ADM implementation [29]. The opacity of automated systems in health care decision-making affects their regulatory approval and poses a problem for trustworthiness and accountability [30]. It has even been shown that the adoption of the right to explanation has been driven, partially, by the hesitancy to use AI applications and limited uptake [31].

As a result, one would think that as the right to explanation has been codified in many countries with hopes of reducing hesitancy and increasing trust around ADM, there would be consensus on the definition. This is not the case. Despite the common use of the phrases “right to explanation,” “explainability,” and “interpretability” with regard to ADM, there is no consensus as to their definitions [32]. Internationally, there are varying norms as to what type of explanation should be provided, why an explanation should be provided, and to whom an explanation is owed [33]. This has brought about a variety of methods used to explain automation, resulting in further ambiguities in what constitutes an explanation [34].

Surprisingly, the elements of a good explanation are not well defined. Instead, many guidelines exist outlining what an explanation should contain. As a result of this varying guidance, there are different ways to categorize explanations. Generally speaking, the guidelines split explanations into two categories: (1) process-based explanations and (2) outcome-based explanations [35]. Process-based explanations demonstrate that the ADM system uses good governance and best practices in its design, whereas outcome-based explanations attempt to unravel the reasoning behind a particular automated decision [36]. The varying significance and typology of explanations is commonly criticized and blamed on legislation that is vague and difficult to put into practice [32]. This is seen in article 15(h), article 22, and recital 71 of the EU GDPR. Article 15 concerns the “Right of access by the data subject” who shall have “the right to obtain from the controller confirmation as to whether or not personal data concerning him or her are being processed, and, where that is the case, access to the personal data and the following information,” and article 15(h) states as follows [23]:

[T]he existence of automated decision-making, including profiling, referred to in Article 22(1) and (4) and, at least in those cases, **meaningful information about the logic involved,** as well as the **significance and the envisaged consequence** of such processing for the data subject.

This phrasing does not set a standard as to what constitutes a good explanation. Rather, it sets an amorphous goal. “Meaningful information” is just another way of saying “explanation.” Furthermore, as ADM models are often not well understood, how is a clinician expected to be able to explain these decisions? A physician is trained to treat patients; any form of a meaningful explanation of an automated decision would require other technical expertise. Much of the literature on the right to explanation has shown that government initiatives to legislate this right provide merely the “illusion of a remedy rather than a burden of proof for legal recourse” [32]. The EU would likely argue that the wording in the GDPR is intended to be a first step; however, EU publications since the GDPR was implemented in 2018 have either barely mentioned explainability or adopt the same language as the GDPR, merely reiterating the right to a “meaningful explanation,” but lacking guidance on what such an explanation should include [37,38].

A similar lack of clarity seems to exist regarding the codification of the right to explanation in the United Kingdom and the United States. As seen with the EU regulation, the focus of legislative initiatives is to foster innovation and competitiveness, with the concepts of transparency and explainability being presented as imprecise protective mechanisms. The United States has mostly relied on self-regulation as seen in the 2019 executive order “Maintaining American Leadership in AI,” a policy designed to ensure AI development that allows growth across multiple sectors [39,40]. Even with the passing of the National AI Initiative Act*,* designed to ensure AI leadership in the United States, the right to explanation remains an amorphous, almost unenforceable, remedy [41]:

Independent regulatory agencies are encouraged, as they deem appropriate, to consider using their full range of authorities to protect American consumers from fraud, discrimination, and threats to privacy and to address other risks that may arise from the use of AI, including risks to financial stability, and to consider rulemaking, as well as emphasizing or clarifying where existing regulations and guidance apply to AI, including clarifying the responsibility of regulated entities to conduct due diligence on and monitor any third-party AI services they use, and emphasizing or clarifying requirements and expectations related to the transparency of AI models and regulated entities' ability to explain their use of AI models.

In the United Kingdom, the situation is no different, where the national data strategy focuses on the responsible growth of AI, without firm guidance as to what constitutes an appropriate explanation [42]. To gain insights into what qualifies as a good explanation for an automated decision, we must, for now, look beyond the legislative frameworks to policy documents. For the sake of clarity, we will focus on the “Ethics Guidelines for Trustworthy AI” by the high-level expert group on artificial intelligence of the EU, outlining conditions that AI should satisfy to ensure that the systems are ethical and responsible, and the “Four Principles of Explainable Artificial Intelligence”’ by the National Institute of Standards and Technology, US Department of Commerce [27,35]. The four principles that are said to be important considerations of what is a good explanation are as follows [35]:

1. Explanation principle—the explanation provides the reasoning behind the decision rendered
2. Meaningful principle—the intended recipient of the explanation understands it
3. Accuracy principle—the explanation accurately describes how the system came to its conclusion
4. Knowledge-limits principle—the explanation clearly highlights the system’s design and knowledge boundaries (ie, the image submitted was blurry; therefore, the system cannot be certain of its decision due to the unclear image)

In addition to the aforementioned four principles, we have an “Assessment List for Trustworthy AI” provided by the EU [27]. This is helpful because it provides a list of questions (eg, why was this automated system deployed in this specific area? Did you analyze your training data?) that can be used to assess the adequacy of an explanation. Both the questions and principles are important to keep top of mind as we move forward to evaluate the possible effects of the right to explanation on the use of ADM in the health care sector.

As is immediately evident, properly implementing an explanation for any automated decision is intensive and starts from the beginning of the program design, regardless of whether the approach to explanation is integrated (ie, the system is transparent and designed to be understandable) or post hoc (ie, the explanation is generated after a prediction or decision is rendered) [43]. However, it is also clear that, given the current guidance on the right to explanation, it still remains an unstructured idea. One country’s sufficient explanation may be completely different from that of another country. The aforementioned principles add detail to what constitutes a good explanation, but they are still vague, even if less vague than the legislation. In the health care setting, this could perhaps be overcome with the development of guidance documents as to what constitutes a good explanation for patients as well as through partnerships with the technology sector to rework ADM systems to provide a form of explanation itself upon which physicians can build.

## The Effect of the Right to Explanation on Health Care Decision-Making

### Overview

The provisions relevant to ADM in health care are in Law 5, which recently came into force. These mirror the provisions on ADM contained in the Private Sector Act and the Public Sector Act, with the only difference being the actor involved—enterprise, public body, or health and social services body, respectively [44]. Section 65 of Law 5 outlines the right to explanation in a health care context as follows [3]:

A body that uses information it holds to render a decision based exclusively on automated processing of the information must inform the person concerned accordingly not later than at the time it informs the person of the decision.

It must also inform the person concerned, at the latter’s request (1) of the information used to render the decision; (2) of the reasons and the principal factors and parameters that led to the decision; and (3) of the right of the person concerned to have the information used to render the decision rectified.

The person concerned must be given the opportunity to submit observations to a member of the body’s personnel or a professional practicing his or her profession within the body who is in a position to review the decision.

The framing of the right to explanation in section 65 of Law 5 could lead to confusion among health care providers and will inevitably need to be elucidated by the courts. The provision writes into law a loophole of sorts that could render the right to explanation irrelevant in clinical contexts. The obligation to inform and explain is solely for decisions “based exclusively on automated processing.” If this is interpreted literally, the right to explanation is avoided as long as a human reviews the decision. As ADM in health care is often used as a second opinion, and human physicians are still involved in the process of diagnosis, this would mean that most everyday uses of ADM in health care would still not require an explanation, even with the passing of Law 5. The uses of ADM currently found in health care that would escape the grasp of section 65 of Law 5 are patient triage tools, diagnostic aids, and telehealth care assistants.

Nonetheless, if the right to explanation exists in these cases, the physician would likely be able to explain basic medical reasons for the diagnosis, as they would be in agreement with the findings of the automated system, but the physician would rarely have the knowledge to explain how the system came to its decision. This would require enumerating how the system was trained, what factors and parameters the system considers, and more. The physician did not train the automated system themselves. The company that produced the automated system would have done so. Furthermore, even the company that produced the device will not understand fully how it functions, unless it is a locked system, meaning it lacks the ability to evolve and learn as it goes. For an unlocked system, asking for a detailed explanation may be asking for the impossible. However, if guided by the principles outlined in the “Four Principles of Explainable Artificial Intelligence,” it may be possible to provide an adequate explanation to a person affected by the automated decision [35]. This explanation would have to come mostly from the automated system itself (ie, as an element of the results it provides) or from the company running the automated system. To be sufficient, it would need to (1) be understandable to the patient, (2) provide reasons for its decision (eg, what parameters are weighed with most importance and a heat map for medical imaging), (3) provide an accurate explanation, and (4) indicate any limits to rendering a decision.

### The Possible Importance of “Based Exclusively on Automated Processing”

#### Overview

Determining when Law 5 will apply is of great importance for understanding the implications of the incorporation of the right to explanation in ADM in health care. If “exclusive” means, as its lay definition would suggest, that the right to explanation is only owed when AI alone is used to render a decision, then Law 5 would not significantly alter a physician’s preexisting duty to inform. However, if the interpretation of Law 5 is more expansive, physicians may be forced to explain each health care decision that was made with the aid of any automated system.

The “exclusively used” interpretation is in line with how the GDPR has been applied, whereas the more expansive interpretation is in line with France’s additions to the GDPR and with proposed Canadian federal legislation, Bill C-27 (the Digital Charter Implementation Act) [45].

#### General Data Protection Regulation

The drafters of the GDPR (Regulation [EU] 2016/679) limited the right to explanation to decisions that are based *solely* on automated processing [23], a framing now reflected in Quebec’s Law 5. The GDPR drafters noted too that these automated decisions must also produce legal effects that significantly affect the person and must be done without any meaningful human intervention [23]. To understand what “exclusively” might mean in the Quebec privacy legislation, it is informative to look for guidance regarding what constitutes solely ADM without meaningful human intervention. To avoid the conclusion that a decision was “based solely on automated processing,” the “Guidelines on Automated Individual Decision-Making and Profiling,” established by the European Commission to protect human rights, state as follows [46]:

The controller cannot...fabricat[e] human involvement. For example, if someone routinely applies automatically generated profiles to individuals without any actual influence on the result, this would still be a decision based solely on automated processing.

To qualify as human involvement, the controller must ensure that any oversight of the decision is meaningful, rather than just a token gesture. It should be carried out by someone who has the authority and competence to change the decision. As part of the analysis, they should consider all the relevant data.

Although this guideline provides some direction, the level of oversight a medical professional must exercise on ADM to reach a meaningful human intervention is still a heavily debated topic [47]. Many academic papers on the topic assert that meaningful intervention needs to be more than routine approval [45]. The European Court of Justice (ECJ) offered additional clarity on the question of what constitutes solely ADM in the case of OQ versus Land Hessen (note that this case, while not in the context of health care, is still relevant as it is expected to clarify what constitutes a decision made solely by ADM. The case concerns an action brough by a “data subject” against a credit score calculation by SCHUFA Holding AG. See OQ v Land Hessen, C‑634/21, [2023] ECLI:EU:C:2023:220 at para 17, 38, 45 [48]). The ECJ issued its judgment in December 2023. Unlike ECJ judgments, which tend to be brief, the opinions of the advocate general (AG) are typically lengthy and delve into the details. In this case, the AG’s opinion, corresponding to the judgment, is much more informative. Notably, the AG’s opinion is not binding [49]. In this case, the AG was of the opinion that the absence of a legal definition of “solely automated” in the EU legislation was indicative of an intentional decision by legislators to keep the definition broad [49]. The AG also focused heavily on the causal link between the automated processing and the final decision. Here, the automated decision under review was a credit score calculation. The AG stated as follows [49]:

[E]ven though human intervention is in principle still possible at that stage of the decision-making process, the decision to enter into a contractual relationship with the data subject “is in practice determined by the score transmitted by credit agencies to such a considerable extent that the score penetrates through the decision of the third-party controller.”

In the words of the ECJ judges, an automated credit score calculation falls into the category of an automated decision and is “without human intervention” [50]. Therefore, at least in the EU context, the test for when a decision is based solely on ADM is whether the automated decision influences the decision of a third party.

If we apply this understanding of solely ADM to the Quebec threshold of exclusively ADM, physicians would be required to demonstrate that ADM was not the pivotal factor in the diagnosis or treatment of a patient to avoid the added explanation obligations of Bill 3. A physician may be able to demonstrate that ADM was not a pivotal factor in their decision if they only look at the automated decision after rendering a decision themselves (ie, using AI as a true second set of eyes). However, this formulaic approach to when ADM is considered in the diagnosis or treatment of a patient may eliminate the streamlining benefits offered by AI (eg, a physician would still need to spend the same amount of time reaching a diagnosis themselves).

#### France

Interestingly, in France, the right to explanation seems to go beyond the right outlined in EU and Quebec legislation. While this bolstered right applies only to administrative decisions made by public bodies, the Loi pour une République numérique (Digital Republic Act) grants the right to explanation when “a decision is taken on the basis of an algorithmic treatment” [51]. France helps us understand that there is a real difference between decisions made with solely or exclusively automated systems and decisions taken on the basis of algorithms. France’s law encompasses more applications of AI than Law 5 and the GDPR do but could be inspiration for the interpretation of Bill C-27 (refer to the next subsection) [52]. This may be explained by the fact that France was the first country in the world to introduce the right to explain into its legislation in the 1970s [20].

#### Bill C-27

Canadian Bill C-27 is currently (as of April 2025) under consideration in the House of Commons, after its second reading. If passed, the bill will revamp private sector privacy laws by introducing three new statutes: the Consumer Privacy Protection Act (CPPA), the Artificial Intelligence and Data Act, and the Personal Information and Data Protection Tribunal Act. Together, these would replace the Personal Information and Protection of Electronic Data Act and serve to modernize and strengthen Canada’s privacy and data protection legal framework [53]. The CPPA will apply to the collection, use, and disclosure of personal information during commercial activity [45]. The benefit of a federal law concerning personal information is that its privacy provisions will also apply to information disclosed interprovincially or internationally [45].

With regard to ADM, the new CPPA expresses the right to be informed of ADM when an “organization has used an automated decision system to make a prediction, recommendation or decision about the individual that could have a significant impact on them” [45]. It also provides the following much-needed definition of what constitutes an automated decision system [45]:

[A]ny technology that “assists or replaces the judgment of human decision-makers” using a rules-based system, regression analysis, predictive analytics, machine learning, deep learning, a neural network or other technique.

It is important to note that as Bill C-27 is not yet law, the definition it proposes is not official from a legislative standpoint but is nonetheless important, as it provides a snapshot of the Canadian legislature context. If this definition is applied in health care in Quebec, a physician using ADM as a second set of eyes would trigger both the obligation to inform and the right to explain, as it would be a case of a technology “assist[ing]” the physician. Furthermore, the inclusion of the phrase “or other technique” to the definition opens the door to have any algorithm that minimally contributed to a decision meet the definition of automated decision system. This could impose obligations on physicians that they are unaware of.

### Duty to Inform in Quebec

The duty to inform requires clinicians to provide patients with the information necessary to make informed decisions about their health care. In Quebec, this duty was first established in jurisprudence in the 1930s [54]. Pursuant to the code of ethics of physicians, a physician must explain “the nature, purpose and possible consequences of the examination, investigation, treatment or research which he plans to carry out” [55]. The goals of the duty to inform are to respect the autonomy of the patient, to promote the respect of the patient, and to ensure that their rights are considered and valued [56]. This duty to inform is reiterated by sections 17 and 65 of Law 5, which lay down the obligations with regard to health and social services information used for ADM in health care. Health and social services information is defined as follows [25]:

1. Information that concerns a patient’s physical or mental health and health determinants
2. Information that concerns any biological material collected from a person in the course of assessment or treatment (including aids that compensate for a disability)
3. Information that concerns health and social services provided to a person
4. Information that was obtained in the exercise of a function under the Public Health Act
5. Information that is any other characteristic determined by government regulation

When this health information is used to “render a decision based *exclusively* on automated processing [ADM],” the person concerned must be informed [25]. Many claim that Law 5 introduces new legal obligations on health bodies and physicians, but we argue that Law 5 merely underscores that the physician’s duty to inform their patients applies equally to situations using ADM as it does to traditional physician-patient interactions; for example, with Law 5, the physician would still be required to explain their diagnosis or prescribing choices to their patients, but they would not be required to explain any automation used to render these decisions so long as automation alone did not make the decision. If the physician also spent meaningful time with the decision, even if less extensively than would have been required to render the same decision without automation, Law 5 does not impose any additional obligations on the physician. If what Quebec is seeking through this legislation is “algorithmic accountability” and transparency [57], the obligation to inform patients regarding ADM reasoning and parameters should not rest solely with physicians and health bodies, nor should it be limited to situations where automation is used exclusively. Physicians are not computer scientists. They are not the designers of these technologies. The computer scientists and the designers of these technologies are much better placed to explain automated decisions.

Nonetheless, if this expectation is placed on physicians, the question remains as to what would change, given their existing obligations. Undoubtedly, requiring the physician to explain how ADM systems arrive at their decisions would restrict the use of ADM in clinician settings. However, if this obligation to explain is limited to decisions made exclusively by ADM systems, the clinician may fulfill their obligations by reviewing the automated decision and determining whether they agree with it based on their training and expertise. The physician would then need to inform the patient that ADM was used as a second opinion. They would still be obliged to enable their patient’s decision-making with as full an understanding of their diagnosis and treatment as possible. However, not much would be required beyond their normal duty to inform. Automation would need to be discussed in a way similar to how a blood test is explained to patients. Physicians typically explain that a blood test will be used for diagnosis purposes, but they do not delve into the details of how a blood test works; in fact, usually, they do not perform the blood test themselves. This raises an important question: if the duties and obligations under Law 5 are restricted to decisions rendered exclusively through ADM, is Bill 3 merely a performative measure by the government to ease the public’s fears concerning AI?

### Effects of Law 5 on a Physician’s Duty to Inform

#### Overview

After understanding the possible scope of Law 5’s application to health care ADM, we must evaluate the effects this legislation may have on the physician’s continuing duty to inform. Physicians using AI to make diagnosis and treatment decisions will likely be required to divulge the use of AI to obtain “free and enlightened consent” as required by their professional order’s code of ethics, regardless of whether it is “solely” used to render a decision or is merely an element of decision-making [55]. To properly inform patients of the involvement of ADM in their care, physicians would need to educate patients about the associated risks (for relevant jurisprudence, see Parenteau c Drolet, 1994 CanLII 5444 (QC CA) [Parenteau] [58] and all cases citing Parenteau, such as Frenette c Clément, 2023 QCCA 109 [59]). As previously discussed, while there is an argument that Law 5 did not introduce this obligation to inform patients of the use of ADM in their diagnosis and treatment so long as it was not being “exclusively” used to render a decision, this argument likely applies only to the further obligation to explain the automated decision. Patients would still be entitled to know that AI is being used in their health care, even if minimally, and physicians are required by their code of ethics to explain the possible risks and benefits associated with their treatment, whether health or technology related.

While informing patients that AI is being used is a first step, the duty to inform also requires physicians to provide their patients with information to understand their diagnosis, the nature and purpose of a proposed treatment, and the risks of the proposed treatment [60]. In a context without ADM, the physician would need to be able to explain to a patient why a certain diagnosis was reached and the treatment options. If ADM was used to arrive at a treatment choice, the physician would need to be able to provide the same level of explanation. However, with Law 5, there is a debate as to whether more would be required of the physician (ie, would the physician need to be able to explain what factors the ADM system considered in making the diagnosis? Would the physician need to describe the algorithm used in decision-making?). Under Law 5, if the right to explanation is interpreted similarly to the GDPR, the physician would be subject to this obligation only if solely ADM was used to render the decision. Given the recent ECJ case law, the decision made exclusively by an ADM system under Law 5 would be defined as a decision where ADM was the primary factor in the physician’s decision. Law 5 and the GDPR both grant physicians discretion to decide when a decision is solely or exclusively automated, as these pieces of legislation do not provide a definition of what falls into this category. This interpretation of Law 5 would render the right to explanation more performative than protective. If the interpretation of Law 5 more closely resembles the rights given to individuals subject to ADM in France, the right to explanation promises to be an onerous addition to physicians’ existing duties.

#### Explaining the Unexplainable

As the physician’s duty to inform has existed in Quebec law for decades, there is ample jurisprudence that clearly outlines what is necessary to discharge the duty. To properly inform a patient, a physician must “address the nature of the intervention and its alternative...as well as important risks of such intervention, taking into consideration the specifics...of each patient” [61]. Law 5 presents hurdles to fulfilling this duty. With the physician’s duty to inform, the physician would need to explain the diagnosis and treatment to their patient and, if the decision was rendered exclusively by an ADM system, they would likely need to inform the patient that an ADM system guided the diagnosis or treatment decision and provide an explanation of the system concerned. The explanation goes beyond informing patients of the use of ADM. It requires physicians to explain how the ADM system reached the decision. This will be near impossible in many cases, especially as automated systems increasingly function as neural networks (eg, deep learning). These systems mimic the human brain, an organ that scientists do not yet fully understand. Furthermore, the number of connections per minute being made in these designed neural networks is untrackable. There will always be new things to understand faster than we can understand them. Finally, an ADM system, by its nature, has elements of its processes that are unexplainable, and the physician may not be aware of them or lack the expertise to explain them. These systems are likely to integrate black boxes into their processes. Black boxes are “internals [that] are either unknown to the observer or known but uninterpretable to humans” [62].

This has led to the fear that unexplainable automated decisions in health care are not entirely accurate due to their very unexplainability and that there is no method for reviewing such decisions. The prediction of coronary heart disease using ADM, for example, has an accuracy rate of approximately 71.5% [63]. If the physician knows that the ADM system being used has a lower rate of accuracy, then, under their preexisting obligations, they would be required to weigh this before presenting the information to the patient. The physician may also choose to tell the patient the rate of accuracy if deemed important for the patient’s proper decision-making. However, placing an extra burden on the physician by requiring them to explain how AI reached a decision is not the answer. The answer lies in the physician’s preexisting obligation to act “to protect and promote the health and well-being of the persons he attends to” [55]. The introduction of technology to health care does not strip away the physician’s duty of care. However, the responsibility should not only fall on physicians. While developers are required to understand the technical dimensions of the AI models they create, health institutions also share responsibility for selecting the AI tools they integrate into medical practice. They are liable for choosing unexplainable models and must establish adequate measures to ensure patient safety and trust [64].

#### Uneasiness With Technology

Law 5’s reiteration of a physician’s duty to inform in situations where ADM assisted with the care of a patient shows legislators’ uneasiness with technology in a space as personal as health care. The legislators do not trust this technology. There is a distaste for the idea that ADM provides an answer that physicians simply need to sign off on (ie, being presented with an answer before thinking of one themselves). They seem to worry that once ADM becomes medical best practice, the physician’s duty to inform their patient about the use of ADM will not be sufficient, at least for the legislators’ taste. This is due to the fact that once ADM becomes common practice, it will not be necessary to discuss its risks in the same way that the risks of a novel therapy or surgery would be discussed. The risks of the use of ADM would be discussed similarly to how the risks of blood tests are discussed—glossed over quickly, with no in-depth discussion [3]. To prevent this from occurring, it can be argued that Law 5 legislates an additional obligation on top of the physician’s duty to inform. The requirements of this obligation to explain do not diminish as a method becomes more frequently used and practiced. The obligation to explain will continue to apply to ADM in health care for as long as ADM is used, regardless of whether it slows down the adoption and advancement of the new technology and regardless of whether this slowdown is actually detrimental to patients and the health care system in its own way.

#### The Unfairness Inherent in Discretionary Decisions

Legislators, while hesitant about the adoption of ADM in health care, have seemingly realized that it is inevitable with the passing of Law 5. While they have outlined rules for the use of ADM in medicine, they have left these rules broad. The issue with broad provisions such as those seen in Law 5 and the GDPR is that these provisions are more likely to have unfair applications. While it is likely that when section 65 of Law 5 should apply (ie, when a decision is considered to have been made “exclusively” with automated processing) will be defined by case law eventually, the interpretation of section 65 for the time being is left to physicians and health bodies [3]. It is conceivable that this will influence the integration of ADM into hospitals and clinics. Hospitals and clinics that adopt a strict definition of “exclusively” will not be as concerned with the new obligations and will be more likely to use ADM more often in their practices. This will make the provision of health services more efficient and accurate. By contrast, establishments that are wary of Law 5 will not be prone to integrate ADM into their practice and will therefore be able to serve fewer patients; in addition, they will not gain the accessibility benefits that AI offers in health care. This will leave the provision of health services more uneven across Quebec than they already are; for example, certain populations will have access to advances in personalized and precision medicine enabled by AI, while others will not.

### Conclusions

In summary, the rapid advancement of technology has brought forth new possibilities and developments in the health care system, including the use of ADM. In health care, ADM uses data, computer systems, and algorithms to make diagnostic and treatment decisions. Its implementation in health care offers several benefits, such as minimizing human error, efficiently processing extensive health datasets, consolidating patient information for predictive results, identifying areas for improvement, and improving accessibility to certain health care procedures.

However, the use of ADM raises concerns regarding patient privacy, the protection of personal information, and transparency in the use of technology. The right to explanation has emerged, from legislators’ standpoint, as a key protection against these concerns, as it entitles patients to an explanation of the algorithm’s results and its impact on their diagnosis and treatment plans. This right has been codified in other legislation around the world, but, in Canada, Quebec is the first jurisdiction to adopt the right to explanation. However, it remains an open question as to whether this right truly provides any meaningful protection for patients affected by automated decisions.

We assert in this paper that the right to explanation introduced by Law 5 and other laws around the world is merely a performative action by legislators that, perhaps due to a lack of understanding of the technologies underpinning ADM, gives the impression of protection but fails to actually do so. While we recognize that Law 5 is an important step forward in including protections related to the right to explanation in today’s reality in the context of AI, the legislations’ definitions remain unclear on how these provisions will be applied in practice. The right to explanation has the potential to be an onerous additional obligation on physicians using ADM in their practice and, as such, serves as a barrier to its adoption and use. Such an onerous obligation may be justified in cases where a new obligation provides true patient protection, but this is not the case with the right to explanation as framed by Law 5.

For Law 5 to evolve into meaningful protection for patients, the law must be applied to all instances involving ADM, rather than only to decisions rendered exclusively by ADM. For this to happen, the vague terminology of Law 5 must be interpreted in a manner similar to the French approach. The courts in Quebec could develop jurisprudence that shifts the interpretation of Law 5 in this direction. In France, legislation beyond the GDPR has strengthened the right to explanation. Quebec often draws inspiration from France; therefore, although the GDPR has served as inspiration for the legislation of the right to explanation worldwide, it is possible for Quebec to follow more closely in France’s footsteps, rather than adopting the minimum requirement of the European approach. If this occurs, especially in the health care setting, then Law 5 would impose burdensome obligations on physicians. The provisions related to the right to explanation would require physicians to explain ADM systems to patients, which, by design, may be unexplainable. To ensure that these additional obligations do not dramatically slow the use of automation in health care settings, the obligations imposed by Law 5 must be imposed on individuals beyond physicians. There would need to be a way to hold software developers and designers responsible for aiding physicians and health care providers in explaining ADM to their patients; for example, the output of these systems could be a diagnosis as well as a rationale for the decision. This would not eliminate the black box inherent to ADM, especially in models that mimic neural networks, but would represent a first step. The way the right to explanation is regulated will affect how responsibility is attributed to different stakeholders, such as clinicians and developers, regarding what information must be disclosed and who will be held accountable if provided or missing information impacts a patient’s health. AI regulation will also need to take into account that errors in outputs can be challenging to trace due to the model’s black box nature. Therefore, it needs to be clear how the right to explanation should be addressed both when developers are building AI models and when clinicians are using the models’ outputs to support decision-making for their patients. While it is understandable that legislators must deal with legal and regulatory questions when a new technology, such as ADM, is introduced, the question arises as to whether the approach taken by Law 5 is the best course of action. Innovative technologies require innovative policies. Developing these laws and policies is no easy task, as it necessitates a deep understanding of complicated, sometimes nearly unexplainable, technologies by legislators. Whatever changes are made to bolster the explanations of ADM, these additional obligations must be carefully thought out, as ADM offers significant benefits in health care and may help to streamline processes, allowing more patients to be seen, diagnosed, and treated. The open question remains: Now that we have legislated the right to explanation, what measures can be implemented to further clarify this right and to ensure that it does not place such burdens on physicians that the adoption of new technologies in health care is stifled?

## Abbreviations

ADM: automated decision-making
AG: advocate general
AI: artificial intelligence
CAD: computer-aided diagnosis
CPPA: Consumer Privacy Protection Act
ECJ: European Court of Justice
EU: European Union
GDPR: General Data Protection Regulation
